# Agent-Based Model with Asymmetric Trading and Herding for Complex Financial Systems

**DOI:** 10.1371/journal.pone.0079531

**Published:** 2013-11-20

**Authors:** Jun-Jie Chen, Bo Zheng, Lei Tan

**Affiliations:** Department of Physics, Zhejiang University, Hangzhou, Zhejiang, China; Tel Aviv University, Israel

## Abstract

**Background:**

For complex financial systems, the negative and positive return-volatility correlations, i.e., the so-called leverage and anti-leverage effects, are particularly important for the understanding of the price dynamics. However, the microscopic origination of the leverage and anti-leverage effects is still not understood, and how to produce these effects in agent-based modeling remains open. On the other hand, in constructing microscopic models, it is a promising conception to determine model parameters from empirical data rather than from statistical fitting of the results.

**Methods:**

To study the microscopic origination of the return-volatility correlation in financial systems, we take into account the individual and collective behaviors of investors in real markets, and construct an agent-based model. The agents are linked with each other and trade in groups, and particularly, two novel microscopic mechanisms, i.e., investors’ asymmetric trading and herding in bull and bear markets, are introduced. Further, we propose effective methods to determine the key parameters in our model from historical market data.

**Results:**

With the model parameters determined for six representative stock-market indices in the world, respectively, we obtain the corresponding leverage or anti-leverage effect from the simulation, and the effect is in agreement with the empirical one on amplitude and duration. At the same time, our model produces other features of the real markets, such as the fat-tail distribution of returns and the long-term correlation of volatilities.

**Conclusions:**

We reveal that for the leverage and anti-leverage effects, both the investors’ asymmetric trading and herding are essential generation mechanisms. Among the six markets, however, the investors’ trading is approximately symmetric for the five markets which exhibit the leverage effect, thus contributing very little. These two microscopic mechanisms and the methods for the determination of the key parameters can be applied to other complex systems with similar asymmetries.

## Introduction

In recent years, the understanding of complex systems has been undergoing rapid development. Financial markets are important examples of complex systems with many-body interactions. The possibility of accessing large amounts of historical financial data has spurred the interest of scientists in various fields, including physics. Plenty of results have been obtained with physical concepts, methods and models [1]–[13].

There are several stylized facts in financial markets. Besides the fat tail in the probability distribution of price returns, it is well-known that the volatilities are long-range correlated in time, which is the so-called volatility clustering [14]. However, our knowledge on the dynamics of the price itself is still limited. Since the auto-correlation of returns is extremely weak [2], [3], nonzero higher-order time correlations become important, especially the lowest-order one among them. In financial markets, this lowest-order nonzero correlation turns out to be the return-volatility correlation, on which we lay emphasis in this paper. In 1976, a negative return-volatility correlation is first discovered by Black [15]. This is the so-called leverage effect, which implies that past negative returns increase future volatilities. The leverage effect is actually observed in various financial systems, such as stock markets, futures markets, bank interest rates and foreign exchange rates [6], [15]–[21]. We have studied about thirty stock-market indices, and all of them exhibit the leverage effect. To the best of our knowledge, the leverage effect exists in almost all stock markets in the world. In Chinese stock markets, however, a positive return-volatility correlation is detected, which is called the anti-leverage effect [6], [19]. This effect is also observed in other economic systems, such as bank interest rates of early years and spot markets of non-ferrous metals.

The leverage and anti-leverage effects are crucial for the understanding of the price dynamics [6], [15], [19], [20], and important for risk management and optimal portfolio choice [22], [23]. However, the origination of the return-volatility correlation is still disputed, even at the macroscopic level [19], [20], [24]–[29]. According to Black, the leverage effect arises because a price drop increases the risk of a company to go bankrupt and leads the stock to fluctuate more. So far, various macroscopic models have been proposed to understand the return-volatility correlation [4], [19], [30]–[33]. The retarded volatility model is an enlightening one, which can produce both the leverage and anti-leverage effects [4]. However, it is a model with only one degree of freedom, and both the initial time series of returns and the function of the feedback return-volatility interaction, are actually input. Hence, the model is phenomenological in essence, and the generation mechanism of the leverage and anti-leverage effects is macroscopic. In very recent years, many researches have been devoted to the return-volatility correlation, but how to produce the return-volatility correlation with a microscopic model remains open.

Agent-based modeling is a powerful simulation technique, which is widely applied in various fields [34]–[41]. More recently, an agent-based model is proposed for reproducing the cumulative distribution of empirical returns and trades in stock markets [41]. It is a outstanding model with key parameters determined from empirical findings rather than from being set artificially. In this paper, we construct an agent-based model with asymmetric trading and herding to explore the microscopic origination of the leverage and anti-leverage effects. In the past decades, although the asymmetric trading and herding behaviors may have been touched macroscopically, they have not been taken into account in the microscopic modeling yet. Especially, we propose effective methods to determine the key parameters in our model from historical market data.

## Methods

To study the microscopic origination of the return-volatility correlation in stock markets, we take into account the individual and collective behaviors of investors, and construct a microscopic model with multi-agent interactions. Further, we determine the key parameters in our model from historical market data rather than from statistical fitting of the results.

Our model is basically built on agents’ daily trading, i.e., buying, selling and holding stocks. Empirical studies indicate that investors make decisions according to the previous stock performance of different time windows [42], which suggests that their horizons of investment vary. This investment horizon is introduced to our model for a better description of agents’ market behavior. Most crucially, two important behaviors of investors are taken into account for understanding the return-volatility correlation.

### 1. Two Important Behaviors of Investors

- Investors’ asymmetric trading in bull and bear markets. There are various definitions of bull and bear markets [43], [44]. The usual definition is that in stock markets, bull and bear markets correspond to the periods of generally increasing and decreasing stock prices respectively [43]. In this paper we adopt this definition, and simply define a market to be bullish on one day if the price return is positive, and bearish if the price return is negative. The asymmetric trading in bull and bear markets is an individual behavior, which is induced by investors’ different trading desire when the price drops and rises. To be more specific, an investor’s willingness to trade is affected by the previous price returns, leading the trading probability to be distinct in bull and bear markets.
- Investors’ asymmetric herding in bull and bear markets. Herding, as one of the collective behaviors, is that investors cluster in groups when making decisions, and these groups can be large in financial markets [45]–[51]. Actually, the herding behavior in bull markets is not the same as that in bear ones [47], [52], [53]. For instance, previous study has shown that in the recent US market, the herding behavior in bear markets appears much more significant than that in bull ones [47]. Generally, investors may cluster more intensively in either bull or bear markets, leading the herding to be asymmetric.

### 2. Microscopic Model with Multi-agent Interactions

The stock price on day 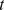 is denoted as 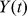, and the logarithmic price return is 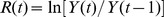. In stock markets, the information for investors is highly incomplete, therefore an agent’s decision of *buy*, *sell* or *hold* is assumed to be random. Since intraday trading is not persistent in empirical trading data [54], we consider that only one trading decision is made by each agent in a single day. In our model, there are 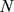 agents, and each operates one share every day. On day 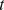, each agent 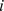 makes a trading decision 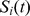,
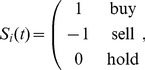
(1)and the probabilities of buy, sell and hold decisions are denoted as 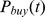, 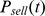 and 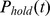, respectively. The price return 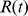 in our model is defined by the difference of the demand and supply of the stock, i.e., the difference between the number of buy agents and sell ones,



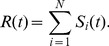
(2)The volatility is defined as the absolute return 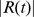.

The investment horizon is introduced since agents’ decision makings are based on the previous stock performance of different time horizons. It has been found that the relative portion 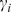 of agents with 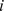 days investment horizon follows a power-law decay, 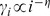 with 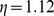 [18]. The maximum investment horizon is denoted as 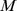, thus 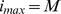. With the condition of 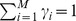, we normalize 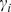 to be 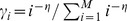. Agents’ trading decisions are made according to the previous price returns. For an agent having investment horizon of 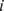 days, 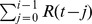 represents a simplified investment basis for decision making on day 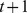. We introduce a weighted average return 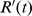 to describe the integrated investment basis of all agents. Taking into account that 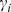 is the weight of 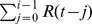, 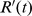 is defined as
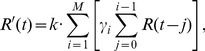
(3)where 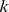 is a proportional coefficient. We set 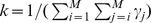, such that 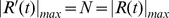 to ensure that the fluctuation scale of 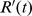 remains consistent with the one of 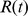 (see Appendix S1). If 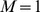, 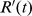 is just identical to 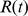. Actually, 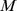 varies from market to market, and from time period to time period for a market. According to Ref. [42], the investment horizons of investors range from a few days to several months. We estimate the maximum investment horizon 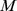 to be 150 in our model. For 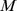 between 50 and 500, the simulated results remain qualitatively robust.

#### (i) Asymmetric trading

In Ref. [41], investors’ probabilities of buy and sell are assumed to be equal, i.e., 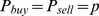, and 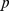 is a constant. In our model, we adopt the value of 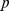 estimated in Ref. [41], 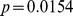. We assume 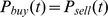 as well, but now 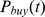 and 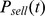 evolve with time since the agents’ trading is asymmetric in bull and bear markets. As the trading probability 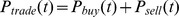, we set its average over time 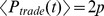. From the investors’ behavior (a) described in Subsec. 1 in Sect. Methods, we define the market performance of the previous 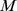 days to be bullish if 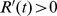, and bearish if 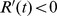. The investors’ asymmetric trading in bull and bear markets gives rise to the distinction between 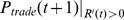 and 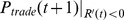. Thus, 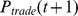 should take the form
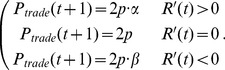
(4)


Here 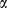 and 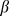 are constants, and 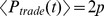 requires 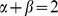, i.e., 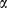 and 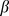 are not independent.

#### (ii) Asymmetric herding

The herding behavior implies that investors can be divided into groups. Here a herding degree 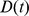 is introduced to quantify the clustering degree of the herding behavior,
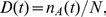
(5)where 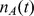 is the average number of agents in each group on day 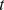. Herding should be related to previous volatilities [46], [55], and we set 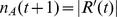. Hence the herding degree on day 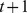 is



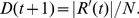
(6)This herding degree is symmetric for 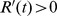 and 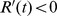. According to the investors’ behavior (b) described in Subsec. 1 in Sect. Methods, however, investors’ herding behaviors in bull and bear markets are asymmetric, i.e., herding is stronger in either bull markets or bear ones. More specifically, 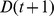 is not symmetric for 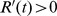 and 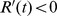, and should be redefined to be
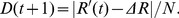
(7)


Here 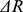 is the degree of asymmetry, and as 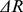 grows, herding becomes more asymmetric. According to Eq. (5), 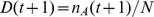. Therefore 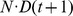 is the average number of agents in a same group. Thus we randomly divide 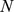 agents into 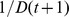 groups on day 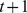. Everyday, the agents in a group make a same trading decision (buy, sell or hold) with the same probability (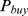, 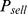 or 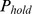).

### 3. Determination of 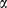 and 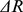


This is the key step in the construction of our model. We emphasize that 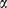 and 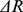 are determined from the historical market data rather than from statistical fitting of the simulated results. Six representative stock-market indices are studied with our model, including the S&P 500, Shanghai, Nikkei 225, FTSE 100, Hangseng and DAX indices. We collect the daily data of closing price and trading volume, both of which are from 1950 to 2012 with 15775 data points for the S&P 500 Index, from 1991 to 2006 with 3928 data points for the Shanghai Index, from 2003 to 2012 with 2367 data points for the Nikkei 225 Index, from 2004 to 2012 with 1801 data points for the FTSE 100 Index, from 2001 to 2012 with 2787 data points for the Hangseng Index and from 2008 to 2012 with 1016 data points for the DAX Index. These data are obtained from “Yahoo! Finance” (http://finance.yahoo.com). For comparison of different time series of returns, the normalized return 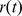 is introduced,
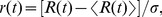
(8)where 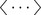 represents the average over time 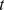, and 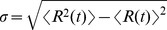 is the standard deviation of 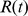.

The stock market is assumed to be bullish if 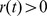, and bearish if 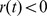. To determine 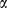, we first define an average trading volume 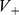 for the bull markets, and 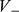 for the bear ones,
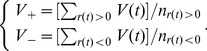
(9)


Here 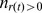 and 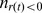 represent the number of positive and negative returns respectively, and 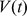 is the trading volume on day 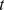. As displayed in Table 1, the ratio 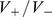 is 1.03 for the S&P 500 Index and 1.21 for the Shanghai Index. In our model, since the average trading volumes for bull markets (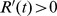) and bear markets (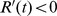) are 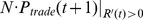 and 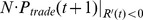, the ratio of these two average trading volumes is
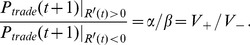
(10)


**Table 1:** The values of 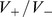, 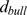, 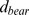, 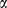, 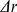 and 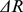 for the six indices.

Index	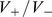	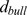	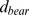	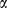	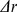	*p*-value	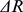
S&P 500 (1950–2012)	1.03	0.993	1.127	1.01±0.01	0.067±0.007	6.7×10^−4^	3
Shanghai (1991–2006)	1.21	0.533	0.447	1.09±0.01	−0.043±0.005	1.0×10^−3^	−2
Nikkei 225 (2003–2012)	1.01	0.729	0.807	1.01±0.01	0.039±0.005	1.5×10^−3^	2
FTSE 100 (2004–2012)	0.98	0.673	0.729	0.99±0.01	0.028±0.003	7.3×10^−4^	2
Hangseng (2001–2012)	1.04	0.966	1.029	1.02±0.2	0.032±0.003	4.4×10^−4^	2
DAX (2008–2012)	0.96	0.797	0.822	0.98±0.02	0.013±0.002	2.9×10^−3^	1

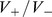
, 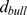 and 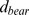 are determined from the historical data for each index. We calculate 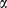 from 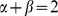 and 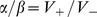, and 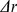 from 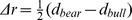. Student’s t-test is performed to analyze the statistical significance of 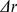. A p-value less than 0.05 is considered statistically significant. We compute 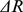 from the linear relation between 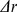 and 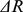 for all these indices. As 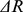 for the Shanghai Index is negative, it is rounded down to the nearest integer, while 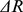 for other indices are positive, and each of them is rounded up to the nearest integer.

Together with the condition 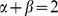, we determine 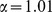 from 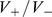 for the S&P 500 Index and 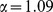 for the Shanghai Index. Table 1 also shows the values of 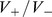 and 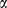 for the Nikkei 225, FTSE 100, Hangseng and DAX indices. Several data series of different time periods are sampled from the historical market data, and the error is given for 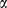 in this table. Student’s *t*-test is performed to analyze the statistical significance for 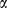 deviating from 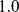, and a *p*-value less than 0.05 is considered statistically significant. The analysis shows that only the value 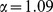 of the Shanghai Index is significantly deviating from 1.0, with the 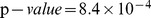. In our simulation, for simplicity, we approximate 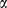 to be 1.0 for the S&P 500, Nikkei 225, FTSE 100, Hangseng and DAX indices, and 1.1 for the Shanghai Index.

Now we turn to 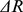. In real markets, herding is related to volatilities [46], [55]. Thus we introduce the average 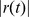 with the weight 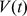 to describe the herding degree in a specific period. Thus the herding degrees of bull markets (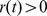) and bear markets (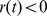) are defined as
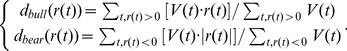
(11)


From empirical findings, the herding degrees of bull and bear stock markets are not equal, i.e., 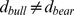. In order to equalize 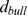 and 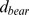, we introduce a shifting to 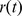, denoted by 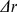, such that 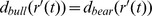 with 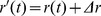. From this definition of 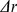, we derive (see Appendix S2)
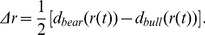
(12)


Thus we obtain 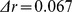 for the S&P 500 Index and 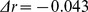 for the Shanghai Index. In our model, we similarly compute the shifting to the time series 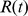, which equalize the herding degree 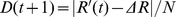 in bull markets (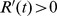) and bear markets (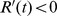). Actually, one may prove that the shifting to 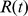 is equivalent to the shifting to 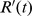 (see Appendix S3). If 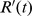 is replaced by 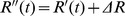, 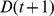 turns into 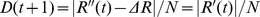, which is symmetric for bull and bear markets. Therefore, 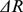 is the shifting to 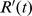, and it is just the shifting to 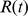.

**Figure 1:**
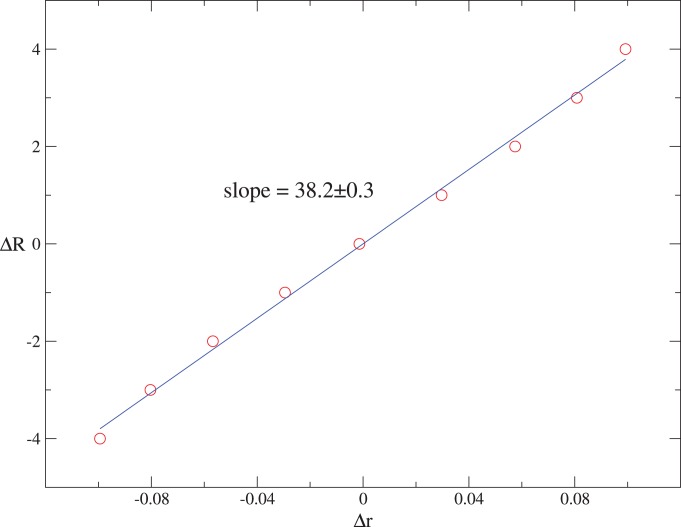
The relation of 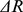 and 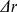. With 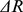 set to be −4, −3, −2, −1, 0, 1, 2, 3 and 4 respectively, time series 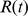 is simulated 100 times for 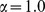. The corresponding 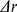 is computed and averaged for each 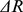. This plot shows a linear relation of 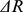 and 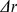, i.e., 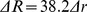, and this result remains robust for 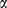 between 0.9 and 1.1.

The time series of returns in different real markets and in our model fluctuate at different levels. For comparison, we normalize the returns with Eq. (8). Similarly, 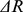, the shifting to returns, should also be normalized to 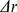. However, in simulating the stock markets with our model, the parameter we need is 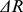. Therefore, we should first derive the relation of 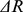 and 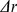. With the normalization of the time series 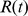, 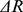 should be normalized to 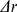,
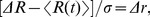
(13)where 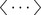 represents the average over time 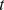, and 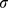 is the standard deviation of 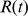. To determine the relation of 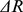 and 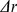, 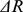 is set to be −4, −3, −2, −1, 0, 1, 2, 3, 4, respectively, and 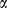 is set to be 1.0 to produce time series 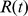. With 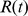 simulated 100 times for each 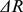, we compute 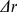 and average the results. As displayed in Fig. 1, the relation of 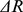 and 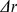 is linear, and 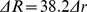. For 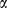 between 0.9 and 1.1, the results remain robust. Thus, we determine 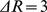 for the simulation of the S&P 500 Index and 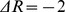 for the simulation of the Shanghai Index. Table 1 shows the values of 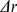 and 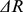, as well as the error of 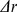, for the Nikkei 225, FTSE 100, Hangseng and DAX indices. Due to the fluctuation of the empirical data, the error of 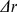 is about 10 percent. Since the sign of 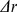 determines that the simulation yields the leverage or anti-leverage effect, we perform Student’s *t*-test to analyze the statistical significance of 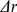, and the corresponding *p*-value is listed in Table 1. A *p*-value less than 0.05 is considered statistically significant.

To further validate the methods for the determination of the key parameters and the simulations for the leverage and anti-leverage effects, eight more indices are studied (see Appendix S4). The simulation of each index correctly produces the leverage or anti-leverage effect.

### 4. Simulation

The number of agents in our simulations is 10000, i.e., 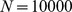. With 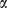 and 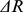 determined for each index, our model produces the time series of returns 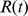 in the following procedure. Initially, the returns of the first 150 time steps are set to be 0. On day 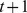, we calculate 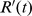 according to Eq. (3), then 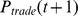 and 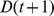 according to Eq. (4) and Eq. (7), respectively. Next, we randomly divide all agents into 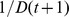 groups. The agents in a group make a same trading decision (buy, sell or hold) with the same probability (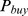, 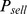 or 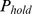). After all agents have made their decisions, we calculate the return 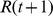 with Eq. (1) and Eq. (2). Repeating this procedure, we obtain the return time series 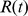 . 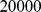 data points of 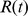 are produced in each simulation, but the first 10000 data points are abandoned for equilibration.

## Results

To describe how past returns affect future volatilities, the return-volatility correlation function 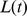 is defined,
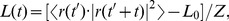
(14)with 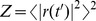 and 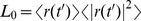 [8]. Here 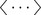 represents the average over time 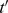.

**Figure 2:**
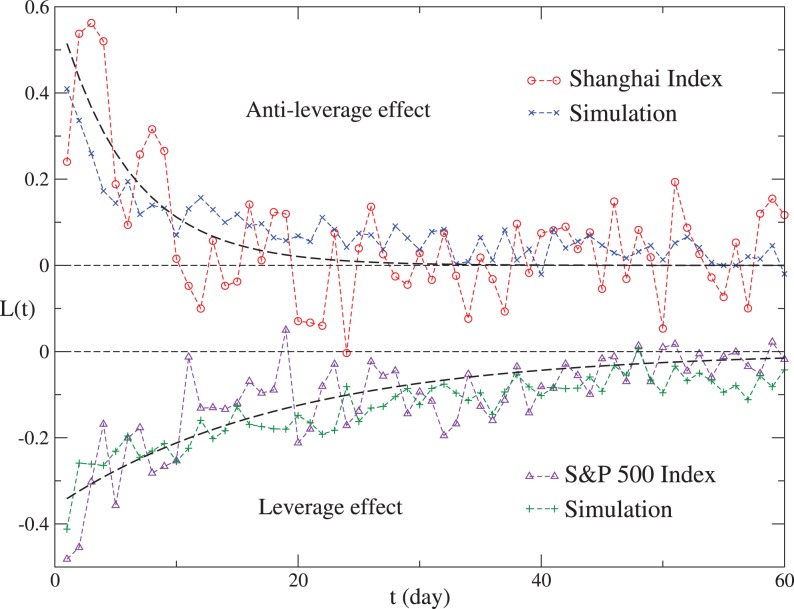
The return-volatility correlation functions for the S&P 500 and Shanghai indices, and for the corresponding simulations. The S&P 500 and Shanghai indices are simulated with 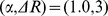 and 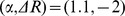, respectively. Dashed lines show an exponential fit 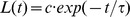 with 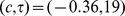 and 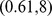 for the S&P 500 Index and the Shanghai Index.

As displayed in Fig. 2, 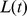 calculated with the empirical data of the S&P 500 Index shows negative values up to at least 15 days, and this is the well-known leverage effect [4], [6], [15]. On the other hand, 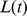 for the Shanghai Index remains positive for about 10 days. That is the so-called anti-leverage effect [6], [19]. Fitting 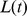 to an exponential form 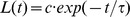, we obtains 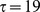 and 8 days for the leverage and anti-leverage effects, respectively. Compared with the short correlating time of the returns, the order of minutes [2], [3], both the leverage and anti-leverage effects are prominent. As the lowest-order nonzero correlations of returns, the leverage and anti-leverage effects are theoretically crucial for the understanding of the price dynamics [6], [15], [19], [20]. In practical application, these effects are important for risk management and optimal portfolio choice [22], [23]. After the time series 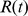 produced in our model is normalized to 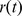, we compute the return-volatility correlation function, and the result is in agreement with that calculated from empirical data on amplitude and duration for both the S&P 500 and Shanghai indices, as shown in Fig. 2. This is the first time that the leverage and anti-leverage effects are produced with a microscopic model.

**Figure 3:**
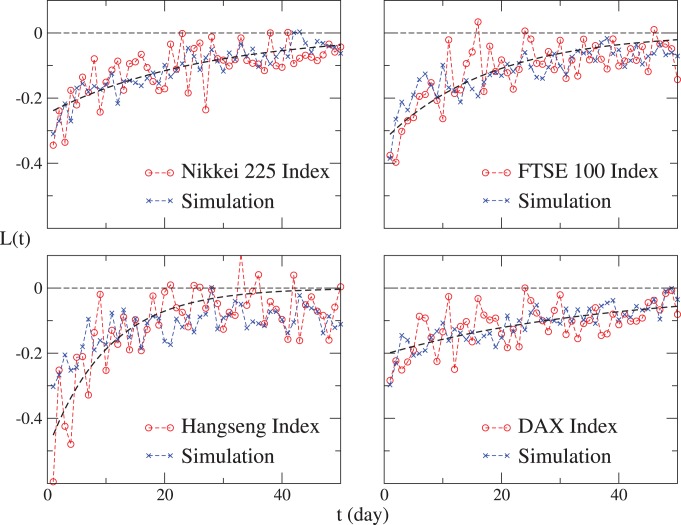
The return-volatility correlation functions for the four indices and the corresponding simulations. The Nikkei 225, FTSE 100, Hangseng and DAX indices are simulated with 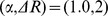, 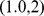, 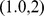 and 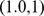, respectively. Dashed lines show an exponential fit 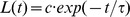 with 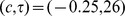 for the Nikkei 225 Index, 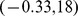 for the FTSE 100 Index, 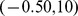 for the Hangseng Index and 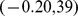 for the DAX Index.

For the Nikkei, FTSE 100, Hangseng and DAX indices, the volume data of early years are not available to us. However, 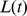 is computed from only price data. In order to reduce the fluctuation of 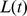, we collect the price data of a longer period, which are from 1984 to 2012 with 7092 data points for the Nikkei 225 Index, from 1984 to 2012 with 7227 data points for the FTSE 100 Index, from 1988 to 2012 with 6181 data points for the Hangseng Index and from 1990 to 2012 with 5514 data points for the DAX Index. As displayed in Fig. 3, 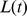 for the simulations is in agreement with that for the corresponding indices. Table 2 shows the values of 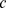 and 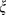 of the exponential fit 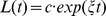 for the six indices and the corresponding simulations. Since 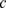 is obviously non-zero, the *p*-value of Student’s *t*-test is only listed for 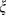.

**Table 2:** The values of 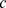 and 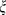 of the exponential fit 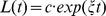 for the six indices and the corresponding simulations.

	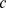	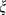	*p*-value
S&P 500	−0.36±0.02	−0.053−0.005	4.5×10^−4^
simulation	−0.30±0.01	−0.032±0.001	5.7×10^−6^
Shanghai	0.61±0.12	−0.133±0.014	6.9×10^−4^
simulation	0.30±0.02	−0.066±0.004	7.9×10^−5^
Nikkei 225	−0.25±0.01	−0.038±0.004	6.9×10^−4^
simulation	−0.27±0.01	−0.042±0.001	1.9×10^−6^
FTSE 100	−0.33±0.03	−0.055±0.007	1.4×10^−3^
simulation	−0.26±0.01	−0.36±0.001	3.6×10^−4^
Hangseng	−0.50±0.06	−0.098±0.001	1.2×10^−3^
simulation	−0.22±0.01	−0.027±0.001	1.1×10^−5^
DAX	−0.20±0.01	−0.026±0.002	2.0×10^−4^
Simulation	−0.22±0.01	−0.31±0.001	6.5×10^−4^

Student’s *t*-test is performed to analyze the statistical significance of 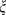. A *p*-value less than 0.05 is considered statistically significant.

**Figure 4:**
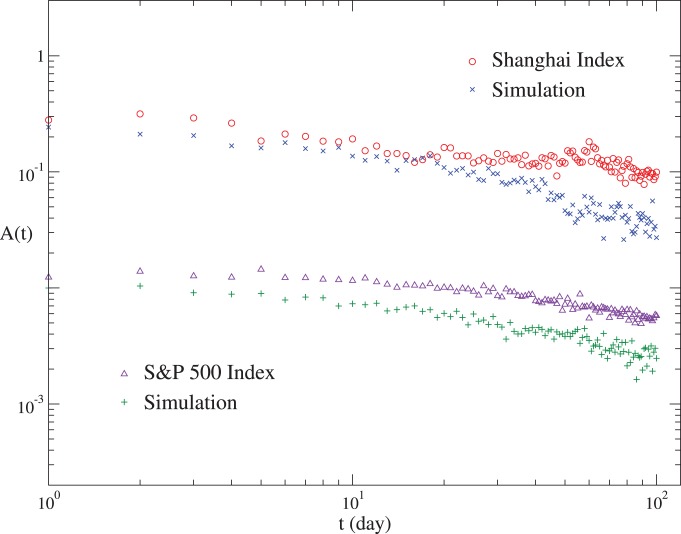
The auto-correlation functions of volatilities for the S&P 500 and Shanghai indices, and for the corresponding simulations. For clarity, the curves for the S&P 500 Index have been shifted down by a factor of 10.

**Figure 5:**
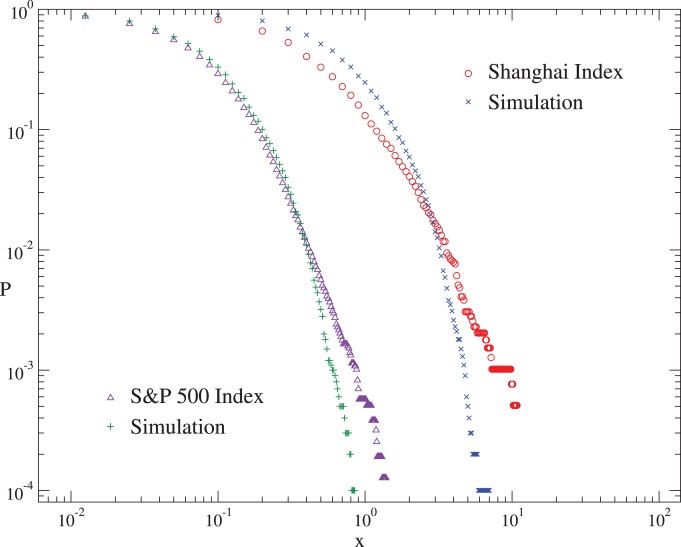
The cumulative distributions of absolute returns for the S&P 500 and Shanghai indices, and for the corresponding simulations. For clarity, the curves for the S&P 500 Index have been shifted left by a factor of 8.5.

Our model also produces other features of the real markets, such as the long-term correlation of volatilities and the fat-tail distribution of the returns. Here we take the S&P 500 and Shanghai indices as examples. The auto-correlation function of volatilities is defined as
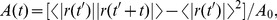
(15)where 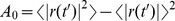 [19], and 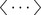 represents the average over time 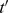. As shown in Fig. 4, 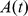 for the simulations is consistent with that for the empirical data. The cumulative distributions 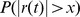 of absolute returns are shown in Fig. 5, where the fat tail in the distribution of empirical returns can be observed in that of the simulated returns as well.

By the definitions, both 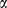 and 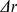 are not dependent on the number of agents (denoted by 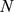) in the model. However, the slope of the linear relation between 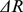 and 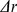 increases with 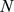. Therefore, the magnitude of 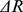 becomes larger as 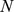 grows. For the simulation results, the amplitude of 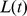 increases with 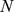, but gradually converges for larger 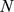 (see Appendix S5). For 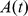 and 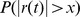, the cases are similar.

## Discussion

In our model, the crucial generation mechanisms of the return-volatility correlation are the agents’ asymmetric trading and herding behaviors in bull and bear markets. Now we discuss how these two mechanisms contribute to the leverage and anti-leverage effects, and which one is more significant. According to Eq. (4) and 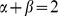, 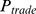 is symmetric about 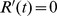 if 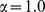, and asymmetric if 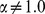. On the other hand, 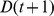 in Eq. (7) is asymmetric about 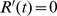 if 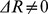. In our model, the S&P 500 and Shanghai indices are simulated with 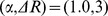 and 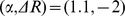, respectively. Therefore, 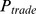 is symmetric in the simulation of the S&P 500 Index, but asymmetric in the simulation of the Shanghai Index. 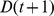 is asymmetric in the simulation of both the S&P 500 and Shanghai indices. With other parts of the model remain unchanged, we consider the following controls: (a) 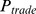 is replaced by a symmetric one in the simulation of the Shanghai Index; (b) 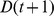 is replaced by a symmetric one in the simulation of both the S&P 500 and Shanghai indices; (c) both 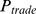 and 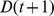 are replaced by the symmetric ones in the simulation of the Shanghai Index.

**Figure 6:**
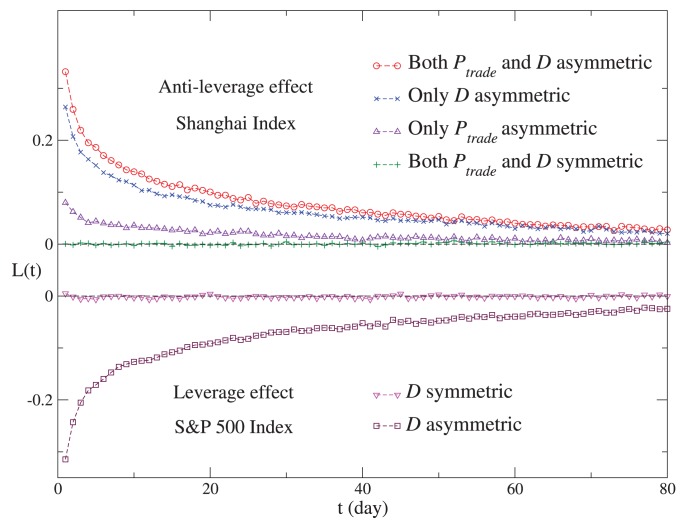
The return-volatility correlation functions for the simulated results of the S&P 500 and Shanghai indices, and for those of the controls. The S&P 500 and Shanghai indices exhibit the leverage and anti-leverage effects, respectively. For the leverage effect, we consider two cases: 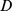 is asymmetric; 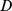 is symmetric. The latter is the control. For the anti-leverage effect, we consider the following cases: both 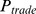 and 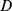 are asymmetric; only 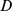 is asymmetric; only 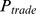 is asymmetric; both 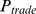 and 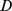 are symmetric. The last three cases are controls. For each case, the simulation is performed for 100 times, and the average 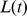 is displayed.

The simulations are performed 100 times for average. We conclude that for the leverage and anti-leverage effects, both the investors’ asymmetric trading and herding are essential generation mechanisms. As displayed in Fig. 6, the anti-leverage effect is weakened significantly and the leverage effect disappears after we replace the asymmetric 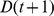 with the symmetric one. On the other hand, the anti-leverage effect recedes after the asymmetric 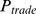 is replaced by the symmetric one. It is worth mentioning that for the five stock markets exhibiting the leverage effect, the S&P 500, Nikkei 225, FTSE 100, Hangseng and DAX, 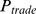 is approximately symmetric, thus contributing very little to the leverage effect. The investors’ asymmetric trading in the Shanghai market may result from the fact that the Shanghai market is an emerging market. Investors are somewhat speculative, and rush for trading as the stock price increases [6].

## Conclusion

Based on investors’ individual and collective behaviors, we construct an agent-based model to investigate how the return-volatility correlation arises in stock markets. In our model, agents are linked with each other and trade in groups. In particular, two novel mechanisms, investors’ asymmetric trading and herding behaviors in bull and bear markets, are introduced. There are four parameters in our model, i.e., 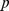, 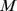, 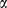 and 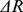. We adopt 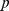 estimated in Ref. [41], and estimate the only tunable parameter 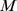 to be 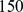. 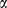 and 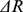, the key parameters, are induced by the asymmetries in trading and herding, respectively. Specifically, we determine 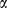 from the ratio of the average trading volume when stock price is rising and that when price is dropping, and 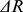 from investors’ different herding degrees in bull and bear markets.

We collect the daily price and volume data of six representative stock-market indices in the world, including the S&P 500, Shanghai, Nikkei 225, FTSE 100, Hangseng and DAX indices. With 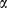 and 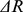 determined for these indices respectively, we obtain the corresponding leverage or anti-leverage effect from the simulation, and the effect is in agreement with the empirical one on amplitude and duration. Other features, such as the long-range auto-correlation of volatilities and the fat-tail distribution of returns, are produced at the same time. Further, it is quantitatively demonstrated in our model that both the investors’ asymmetric trading and herding are essential generation mechanisms for the leverage and anti-leverage effects at the microscopic level. However, the investors’ trading is approximately symmetric for the five stock markets exhibiting the leverage effect, thus contributing very little to the effect. These two microscopic mechanisms and the methods for the determination of 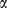 and 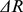 can also be applied to other complex economic systems with similar asymmetries in individual and collective behaviors, e.g., to futures markets, bank interest rates, foreign exchange rates and spot markets of non-ferrous metals.

## Supporting Information

Appendix S1
**Derivation of** 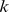.(PDF)

Appendix S2
**Derivation of** 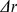.(PDF)

Appendix S3
**Equivalence of the shifting to** 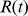 **and that to** 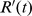 **.**
(PDF)

Appendix S4
**The values of** 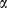 **,** 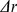 **and** 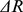 **for eight more indices.**
(PDF)

Appendix S5
**How** 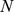 **affects the model parameters and simulation results.**
(PDF)

## References

[1] Mantegna RN, Stanley HE (1995) Scaling behaviour in the dynamics of an economic index. Nature 376: 46–49.

[2] Gopikrishnan P, Plerou V, Amaral LAN, Meyer M, Stanley HE (1999) Scaling of the distribution of fluctuations of financial market indices. Phys Rev E 60: 5305. 10.1103/physreve.60.5305 11970400

[3] Liu Y, Gopikrishnan P, Cizeau P, Meyer M, Peng CK, et al (1999) Statistical properties of the volatility of price fluctuations. Phys Rev E 60: 1390. 10.1103/physreve.60.1390 11969899

[4] Bouchaud JP, Matacz A, Potters M (2001) Leverage effect in financial markets: the retarded volatility model. Phys Rev Lett 87: 228701. 11736431 10.1103/PhysRevLett.87.228701

[5] Gabaix X, Gopikrishnan P, Plerou V, Stanley HE (2003) A theory of power-law distributions in financial market fluctuations. Nature 423: 267–270. 12748636 10.1038/nature01624

[6] Qiu T, Zheng B, Ren F, Trimper S (2006) Return-volatility correlation in financial dynamics. Phys Rev E 73: 065103. 10.1103/PhysRevE.73.065103 16906892

[7] Shen J, Zheng B (2009) Cross-correlation in financial dynamics. Europhys Lett 86: 48005.

[8] Qiu T, Zheng B, Chen G (2010) Financial networks with static and dynamic thresholds. New J Phys 12: 043057.

[9] Zhao L, Yang G, Wang W, Chen Y, Huang JP, et al (2011) Herd behavior in a complex adaptive system. Proc Natl Acad Sci 108: 15058–15063. 21876133 10.1073/pnas.1105239108 PMC3174612

[10] Preis T, Schneider JJ, Stanley HE (2011) Switching processes in financial markets. Proc Natl Acad Sci 108: 7674–7678. 21521789 10.1073/pnas.1019484108 PMC3093477

[11] Zhou WX, Mu GH, Chen W, Sornette D (2011) Investment strategies used as spectroscopy of financial markets reveal new stylized facts. PLOS One 6: e24391. 21935403 10.1371/journal.pone.0024391 PMC3173398

[12] Jiang XF, Zheng B (2012) Anti-correlation and subsector structure in financial systems. Europhys Lett 97: 48006.

[13] Jiang XF, Chen TT, Zheng B (2013) Time-reversal asymmetry in financial systems. Physica A 392: 5369–5375.

[14] Yamasaki K, Muchnik L, Havlin S, Bunde A, Stanley HE (2005) Scaling and memory in volatility return intervals in financial markets. Proc Natl Acad Sci 102: 9424–9428. 15980152 10.1073/pnas.0502613102 PMC1166612

[15] Black F (1976) Studies of stock price volatility changes. Alexandria: Proceedings of the 1976 Meetings of the American Statistical Association, Business and Economical Statistics Section, 177–181.

[16] Engle RF, Patton AJ (2001) What good is a volatility model. Quantitative finance 1: 237–245.

[17] Bollerslev T, Litvinova J, Tauchen G (2006) Leverage and volatility feedback effects in highfrequency data. Journal of Financial Econometrics 4: 353–384.

[18] Qiu T, Zheng B, Ren F, Trimper S (2007) Statistical properties of German DAX and Chinese Indices. Physica A 378: 387–398.

[19] Shen J, Zheng B (2009) On return-volatility correlation in financial dynamics. Europhys Lett 88: 28003. 10.1103/PhysRevE.73.065103 16906892

[20] Park BJ (2011) Asymmetric herding as a source of asymmetric return volatility. Journal of Banking & Finance 35: 2657–2665.

[21] Preis T, Kenett DY, Stanley HE, Helbing D, Ben-Jacob E (2012) Quantifying the behavior of stock correlations under market stress. Scientific reports 2: 752. 23082242 10.1038/srep00752 PMC3475344

[22] Bouchaud JP, Potters M (2001) More stylized facts of financial markets: leverage effect and downside correlations. Physica A 299: 60–70.

[23] Buraschi A, Porchia P, Trojani F (2010) Correlation risk and optimal portfolio choice. The Journal of Finance 65: 393–420.

[24] Haugen RA, Talmor E, Torous WN (1991) The effect of volatility changes on the level of stock prices and subsequent expected returns. J Financ 46: 985–1007.

[25] Bekaert G, Wu G (2000) Asymmetric volatility and risk in equity markets. Rev Financ Stud 13: 1–42.

[26] Giraitis L, Leipus R, Robinson PM, Surgailis D (2004) Larch, leverage, and long memory. Journal of Financial Econometrics 2: 177–210.

[27] Ahlgren PTH, Jensen MH, Simonsen I, Donangelo R, Sneppen K (2007) Frustration driven stock market dynamics: leverage effect and asymmetry. Physica A 383: 1–4.

[28] Roman HE, Porto M, Dose C (2008) Skewness, long-time memory, and non-stationarity: application to leverage effect in financial time series. EPL 84: 28001.

[29] Li J (2011) Volatility components, leverage effects, and the return–volatility relations. Journal of Banking & Finance 35: 1530–1540.

[30] Baillie RT, Bollerslev T, Mikkelsen HO (1996) Fractionally integrated generalized autoregressive conditional heteroskedasticity. J Econom 74: 3–30.

[31] Tang TL, Shieh SJ (2006) Long memory in stock index futures markets: a value-at-risk approach. Physica A 366: 437–448.

[32] Masoliver J, Perelló J (2006) Multiple time scales and the exponential Ornstein–Uhlenbeck stochastic volatility model. Quantitative Finance 6: 423–433.

[33] Ruiz E, Veiga H (2008) Modelling long-memory volatilities with leverage effect: A-LMSV versus FIEGARCH. Comput Stat Data Anal 52: 2846–2862.

[34] Giardina I, Bouchaud JP, Mézard M (2001) Microscopic models for long ranged volatility correlations. Physica A 299: 28–39.

[35] Challet D, Marsili M, Zhang YC (2001) Stylized facts of financial markets and market crashes in minority games. Physica A 294: 514–524.

[36] Bonabeau E (2002) Agent-based modeling: methods and techniques for simulating human systems. Proc Natl Acad Sci 99: 7280–7287. 12011407 10.1073/pnas.082080899 PMC128598

[37] Evans TP, Kelley H (2004) Multi-scale analysis of a household level agent-based model of landcover change. Journal of Environmental Management 72: 57–72. 15246574 10.1016/j.jenvman.2004.02.008

[38] Ren F, Zheng B, Qiu T, Trimper S (2006) Minority games with score-dependent and agent-dependent payoffs. Physical Review E 74: 041111. 10.1103/PhysRevE.74.041111 17155026

[39] Farmer JD, Foley D (2009) The economy needs agent-based modelling. Nature 460: 685–686. 19661896 10.1038/460685a

[40] Schwarz N, Ernst A (2009) Agent-based modeling of the diffusion of environmental innovations: an empirical approach. Technological forecasting and social change 76: 497–511.

[41] Feng L, Li B, Podobnik B, Preis T, Stanley HE (2012) Linking agent-based models and stochastic models of financial markets. Proc Natl Acad Sci 109: 8388–8393. 22586086 10.1073/pnas.1205013109 PMC3365211

[42] Menkhoff L (2010) The use of technical analysis by fund managers: international evidence. Journal of Banking & Finance 34: 2573–2586.

[43] Pagan AR, Sossounov KA (2002) A simple framework for analysing bull and bear markets. Journal of Applied Econometrics 18: 23–46.

[44] Jansen DW, Tsai CL (2010) Monetary policy and stock returns: financing constraints and asymmetries in bull and bear markets. Journal of Empirical Finance 17: 981–990.

[45] Eguiluz VM, Zimmermann MG (2000) Transmission of information and herd behavior: an application to financial markets. Physical Review Letters 85: 5659–5662. 11136071 10.1103/PhysRevLett.85.5659

[46] Cont R, Bouchaud JP (2000) Herd behavior and aggregate fluctuations in financial markets. Macroeconomic Dyn 4: 170–196.

[47] Hwang S, Salmon M (2004) Market stress and herding. Journal of Empirical Finance 11: 585–616.

[48] Zheng B, Qiu T, Ren F (2004) Two-phase phenomena, minority games, and herding models. Physical Review E 69: 046115–1. 10.1103/PhysRevE.69.046115 15169077

[49] Kenett DY, Shapira Y, Madi A, Bransburg-Zabary S, Gur-Gershgoren G, et al (2011) Index cohesive force analysis reveals that the US market became prone to systemic collapses since 2002. PLOS One 6: e19378. 21556323 10.1371/journal.pone.0019378 PMC3083438

[50] Kenett DY, Preis T, Gur-Gershgoren G, Ben-Jacob E (2012) Quantifying meta-correlations in financial markets. Europhys Lett 99: 38001.

[51] Kenett DY, Ben-Jacob E, Stanley HE, Gur-Gershgoren G (2013) How high frequency analysis affects a market index. Scientific Reports 3: 2110. 23817553 10.1038/srep02110 PMC3743071

[52] Kim KA, Nofsinger JR (2005) Institutional herding, business groups, and economic regimes: evidence from Japan. The Journal of Business 78: 213–242.

[53] Walter A, MoritzWeber F (2006) Herding in the German mutual fund industry. European Financial Management 12: 375–406.

[54] Eisler Z, Kertesz J (2007) Liquidity and the multiscaling properties of the volume traded on the stock market. Europhys Lett 77: 28001.

[55] Blasco N, Corredor P, Ferreruela S (2012) Does herding affect volatility? implications for the spanish stock market. Quantitative Finance 12: 311–327.

