# Supplementary material for: Agent-Based Model with Asymmetric Trading and Herding for Complex Financial Systems
Source: PLoS One. 2013 Nov 20;8(11):e79531. doi: 10.1371/journal.pone.0079531 (PMC3835857; doi:10.1371/journal.pone.0079531)
Supplement: Appendix S1 — Derivation of . (PDF) [file pone.0079531.s001.pdf]

Appendix S1

Derivation of k

Here, we derive the coefficient k in Eq. (3) in the main body of the paper by the condition $|R'(t)|_{max} = N = |R(t)|_{max}$. According to Eq. (3), $R'(t)$ is defined as

$$R'(t) = k \cdot \sum_{i=1}^{M} \left[\gamma_i \sum_{j=0}^{i-1} R(t-j) \right],$$

so we have

$$\begin{aligned} R'(t) = k \cdot \{ & \gamma_1 \cdot R(t) + \gamma_2 \cdot [R(t) + R(t-1)] + \cdots + \\ & \gamma_M \cdot [R(t) + R(t-1) + \cdots + R(t-(M-1))]\} \\ = k \cdot [& (\gamma_1 + \gamma_2 + \cdots + \gamma_M) \cdot R(t) + (\gamma_2 + \gamma_3 + \cdots + \gamma_M) \cdot R(t-1) + \cdots + \\ & \gamma_M \cdot R(t-(M-1))]. \end{aligned}$$

Thus, the maximum of $R'(t)$ is

$$\begin{aligned} |R'(t)|_{max} = k \cdot [& (\gamma_1 + \gamma_2 + \cdots + \gamma_M) \cdot |R(t)|_{max} + \\ & (\gamma_2 + \gamma_3 + \cdots + \gamma_M) \cdot |R(t-1)|_{max} + \cdots + \gamma_M \cdot |R(t-(M-1))|_{max}] \end{aligned}$$

Since $|R(t)|_{max} = n$ for each t, we have

$$\begin{aligned} |R'(t)|_{max} &= k \cdot [(\gamma_1 + \gamma_2 + \cdots + \gamma_M) \cdot n + (\gamma_2 + \gamma_3 + \cdots + \gamma_M) \cdot n + \cdots + \gamma_M \cdot n] \\ &= k \cdot (\sum_{j=1}^{M} \gamma_j + \sum_{j=2}^{M} \gamma_j + \cdots + \sum_{j=M}^{M} \gamma_j) \cdot n \\ &= k \cdot (\sum_{i=1}^{M} \sum_{j=i}^{M} \gamma_j) \cdot n. \end{aligned}$$

We require $|R'(t)|_{max} = n$, thus k is

$$k = 1/(\sum_{i=1}^{M} \sum_{j=i}^{M} \gamma_j).$$
