# Supplementary material for: Agent-Based Model with Asymmetric Trading and Herding for Complex Financial Systems
Source: PLoS One. 2013 Nov 20;8(11):e79531. doi: 10.1371/journal.pone.0079531 (PMC3835857; doi:10.1371/journal.pone.0079531)
Supplement: Appendix S2 — Derivation of . (PDF) [file pone.0079531.s002.pdf]

Appendix S2

Derivation of Δr

According to Eq. (11) in the main body of the paper, the herding degree of bull markets ($r(t) > 0$) and bear markets ($r(t) < 0$) are defined as

$$\begin{cases} d_{bull}(r(t)) = \sum_{t,r(t)>0}[V(t)\cdot r(t)]/\sum_{t,r(t)>0}V(t) \\ d_{bear}(r(t)) = \sum_{t,r(t)<0}[V(t)\cdot |r(t)|]/\sum_{t,r(t)<0}V(t) \end{cases}.$$

We introduce a shifting of $r(t)$, denoted by Δr, such that $d_{bull}(r'(t)) = d_{bear}(r'(t))$ with $r'(t) = r(t) + \Delta r$. With $r(t)$ replaced by $r'(t)$ in the above equation, we have

$$\begin{cases} d_{bull}(r'(t)) = \sum_{t,r'(t)>0}\{V(t)\cdot [r(t)+\Delta r]\}/\sum_{t,r'(t)>0}V(t) \\ d_{bear}(r'(t)) = \sum_{t,r'(t)<0}[V(t)\cdot |r(t)+\Delta r|]/\sum_{t,r'(t)<0}V(t) \end{cases}.$$

Δr is first assumed to be small, and this is verified from the practical calculation. Hence, $r'(t) > 0$ and $r'(t) < 0$ are approximately $r(t) > 0$ and $r(t) < 0$, respectively. Therefore,

$$\begin{cases} d_{bull}(r'(t)) = \sum_{t,r(t)>0}\{V(t)\cdot [r(t)+\Delta r]\}/\sum_{t,r(t)>0}V(t) \\ d_{bear}(r'(t)) = \sum_{t,r(t)<0}[V(t)\cdot |r(t)+\Delta r|]/\sum_{t,r(t)<0}V(t) \end{cases}.$$

Thus, we have

$$d_{bull}(r'(t)) = \sum_{t,r(t)>0}[V(t)\cdot r(t)]/\sum_{t,r(t)>0}V(t) + \Delta r,$$

and

$$\begin{aligned} d_{bear}(r'(t)) &= -\sum_{t,r(t)<0}[V(t)\cdot r(t)]/\sum_{t,r(t)<0}V(t) - \Delta r \\ &= \sum_{t,r(t)<0}[V(t)\cdot |r(t)|]/\sum_{t,r(t)<0}V(t) - \Delta r. \end{aligned}$$

Inserting the above two equations into $d_{bull}(r'(t)) = d_{bear}(r'(t))$, we have

$$\sum_{t,r(t)>0}[V(t)\cdot r(t)]/\sum_{t,r(t)>0}V(t) - \sum_{t,r(t)<0}[V(t)\cdot |r(t)|]/\sum_{t,r(t)<0}V(t) + 2\Delta r = 0.$$

Therefore,

$$\begin{aligned} \Delta r &= \frac{1}{2}\left\{\frac{\sum_{t,r(t)<0}[V(t)\cdot |r(t)|]}{\sum_{t,r(t)<0}V(t)} - \frac{\sum_{t,r(t)>0}[V(t)\cdot r(t)]}{\sum_{t,r(t)>0}V(t)}\right\} \\ &= \frac{1}{2}[d_{bear}(r(t)) - d_{bull}(r(t))]. \end{aligned}$$
