# Supplementary material for: Agent-Based Model with Asymmetric Trading and Herding for Complex Financial Systems
Source: PLoS One. 2013 Nov 20;8(11):e79531. doi: 10.1371/journal.pone.0079531 (PMC3835857; doi:10.1371/journal.pone.0079531)
Supplement: Appendix S3 — Equivalence of the shifting to and that to . (PDF) [file pone.0079531.s003.pdf]

Appendix S3

Equivalence of the shifting to $R(t)$ and that to $R'(t)$

According to Eq. (3) in the main body of the paper, $R'(t)$ is defined as

$$R'(t) = k \cdot \sum_{i=1}^{M} \left[\gamma_i \sum_{j=0}^{i-1} R(t-j) \right].$$

To prove that the shifting to $R(t)$ is equivalent to the shifting to $R'(t)$, it's identical to prove the equation:

$$R'(t) + c = k \cdot \sum_{i=1}^{M} \left\{ \gamma_i \sum_{j=0}^{i-1} [R(t-j) + c] \right\},$$

where c is a constant. The term on the right-hand side of this equation is

$$\begin{aligned} k \cdot \sum_{i=1}^{M} \left\{ \gamma_i \sum_{j=0}^{i-1} [R(t-j) + c] \right\} &= k \cdot \sum_{i=1}^{M} \left[\gamma_i \sum_{j=0}^{i-1} R(t-j) + \gamma_i \sum_{j=0}^{i-1} c \right] \\ &= k \cdot \sum_{i=1}^{M} \left[\gamma_i \sum_{j=0}^{i-1} R(t-j) \right] + k \cdot \sum_{i=1}^{M} (\gamma_i \cdot i \cdot c) \\ &= R'(t) + k \cdot \sum_{i=1}^{M} (\gamma_i \cdot i \cdot c). \end{aligned}$$

Since $k = 1/(\sum_{i=1}^{M} \sum_{j=i}^{M} \gamma_j)$, we have

$$\begin{aligned} k \cdot \sum_{i=1}^{M} \left\{ \gamma_i \sum_{j=0}^{i-1} [R(t-j) + c] \right\} &= R'(t) + \frac{\sum_{i=1}^{M} (\gamma_i \cdot i \cdot c)}{\sum_{i=1}^{M} \sum_{j=i}^{M} \gamma_j} \\ &= R'(t) + c \cdot \frac{\sum_{i=1}^{M} (i \cdot \gamma_i)}{(\gamma_1 + \gamma_2 + \cdots + \gamma_M) + (\gamma_2 + \cdots + \gamma_M) + \cdots + (\gamma_{M-1} + \gamma_M) + \gamma_M} \\ &= R'(t) + c \cdot \frac{\sum_{i=1}^{M} (i \cdot \gamma_i)}{\gamma_1 + 2\gamma_2 + 3\gamma_3 + \cdots + M\gamma_M} \\ &= R'(t) + c. \end{aligned}$$

Therefore, the shifting to $R(t)$ is equivalent to the shifting to $R'(t)$.
