# Supplementary material for: Agent-Based Model with Asymmetric Trading and Herding for Complex Financial Systems
Source: PLoS One. 2013 Nov 20;8(11):e79531. doi: 10.1371/journal.pone.0079531 (PMC3835857; doi:10.1371/journal.pone.0079531)
Supplement: Appendix S4 — The values of , and for eight more indices. (PDF) [file pone.0079531.s004.pdf]

Appendix S4

The values of α, Δr and ΔR for eight more indices

The values of V_+/V_-, d_{bull}, d_{bear}, α, Δr and ΔR for eight more indices are displayed below in Table 1. In the simulation for all these indices, the parameter α is set to be 1.0. As described in the main body of the paper, a negative ΔR in the simulation produces the anti-leverage effect, while a positive one yields the leverage effect. These results are in agreement with the empirical ones, and further confirm the methods for the determination of the key parameters and the simulations for the leverage and anti-leverage effects.

Table 1: **The values of V_+/V_-, d_{bull}, d_{bear}, α, Δr and ΔR for the eight indices.** V_+/V_-, d_{bull} and d_{bear} are determined from the historical data for each index. We calculate α from $\alpha + \beta = 2$ and $\alpha/\beta = V_+/V_-$, and Δr from $\Delta r = \frac{1}{2}(d_{bear} - d_{bull})$. Student's t-test is performed to analyze the statistical significance of Δr. A p-value less than 0.05 is considered statistically significant. We compute ΔR from the linear relation between Δr and ΔR for all these indices. As ΔR for the London gold spot price index is negative, it is rounded down to the nearest integer, while ΔR for other indices are positive, and each of them is rounded up to the nearest integer.

Price Index	V_+/V_-	d_{bull}	d_{bear}	α	Δr	p-value	ΔR
Australia stock (2003-2012)	1.00	0.739	0.824	1.00 ± 0.01	0.043 ± 0.004	4.2×10^{-4}	2
Belgium stock (2004-2012)	0.92	0.889	0.943	0.96 ± 0.02	0.027 ± 0.005	5.7×10^{-3}	2
France stock (2004-2012)	0.94	0.800	0.853	0.97 ± 0.01	0.027 ± 0.004	2.5×10^{-3}	2
India stock (2003-2012)	0.98	0.769	0.913	0.99 ± 0.01	0.072 ± 0.007	5.0×10^{-4}	3
Ireland stock (2004-2012)	1.02	0.731	0.817	1.01 ± 0.01	0.043 ± 0.004	4.2×10^{-4}	2
Maxico stock (2001-2012)	1.02	0.785	0.814	1.01 ± 0.01	0.015 ± 0.002	1.7×10^{-3}	1
NewZealand stock (2004-2012)	1.02	0.720	0.803	1.01 ± 0.02	0.042 ± 0.004	4.7×10^{-4}	2
London gold spot (1996-2004)	0.95	0.792	0.681	0.97 ± 0.01	-0.056 ± 0.004	1.5×10^{-4}	-3
