# Supplementary material for: Agent-Based Model with Asymmetric Trading and Herding for Complex Financial Systems
Source: PLoS One. 2013 Nov 20;8(11):e79531. doi: 10.1371/journal.pone.0079531 (PMC3835857; doi:10.1371/journal.pone.0079531)
Supplement: Appendix S5 — How affects the model parameters and simulation results. (PDF) [file pone.0079531.s005.pdf]

Appendix S5

How N affects the model parameters and simulation results

Since α is determined from $\alpha + \beta = 2$ and $\alpha/\beta = V_+/V_-$, and Δr from $\Delta r = \frac{1}{2}(d_{bear} - d_{bull})$, both α and Δr are not dependent on the number of agents (denoted by N). On the other hand, ΔR is calculated from the linear relation between ΔR and Δr, which is obtained through the simulation of the model. As shown below in Table 1, the slope of the linear relation increases as N grows, and it leads to a larger magnitude of ΔR for larger N. In the main body of the paper, for simplicity, ΔR is approximated to an integer in the simulation for each index. To investigate how N affects the simulation results, however, ΔR should be more accurate, and it is now corrected to one decimal place. The influence of N on $L(t)$ is displayed in Fig. 1. The amplitude of $L(t)$ increases with N, but gradually converges for larger N. For $A(t)$ and $P(|r(t)| > x)$, the cases are similar.

Table 1: **The values of ΔR and the relations between ΔR and Δr for different N for the S&P 500 and Shanghai indices.**

N	relation of ΔR and Δr	S&P 500 Index		Shanghai Index	
		Δr	ΔR	Δr	ΔR
1250	$\triangle R = 12.4\Delta r$	0.067	0.8	-0.043	-0.5
2500	$\triangle R = 17.6\Delta r$	0.067	1.2	-0.043	-0.8
5000	$\triangle R = 25.4\Delta r$	0.067	1.7	-0.043	-1.1
10000	$\triangle R = 38.2\Delta r$	0.067	2.6	-0.043	-1.6
20000	$\triangle R = 61.6\Delta r$	0.067	4.1	-0.043	-2.6

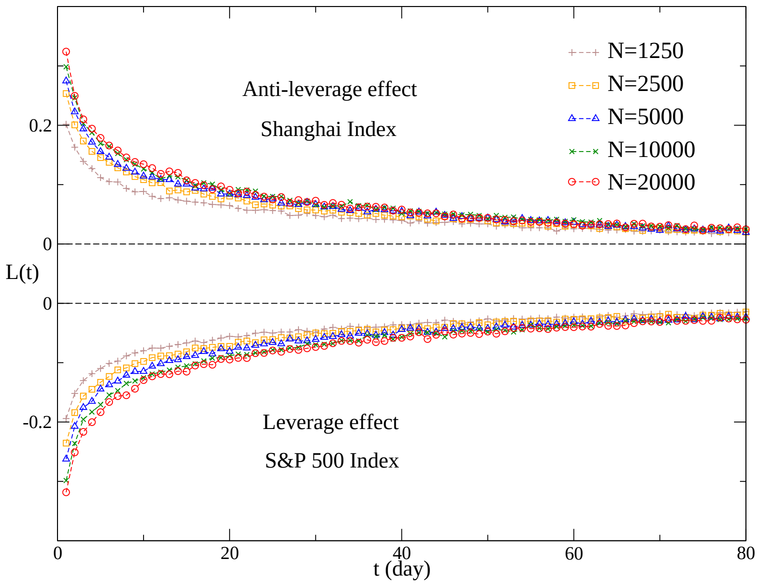

Figure 1: $L(t)$ **from the simulations with different N for the S&P 500 and Shanghai indices.** The S&P 500 Index exhibits the leverage effect, and the Shanghai Index shows the anti-leverage effect. For each N, the simulation is performed 100 times for average.
